# Supplementary material for: Altered microRNA Transcriptome in Cultured Human Liver Cells upon Infection with Ebola Virus
Source: Int J Mol Sci. 2021 Apr 6;22(7):3792. doi: 10.3390/ijms22073792 (PMC8038836; doi:10.3390/ijms22073792)
Supplement: Supplementary file 1 [file ijms-22-03792-s001.zip › Supplementary_File/D_ KEGG_Analysis_Results/16-30nt_pathway_Mayinga-24h-Huh7_vs_Control-24h-Huh7_up.mature_mirna_targets/hsa_pathwayResult.html]

| PathwayID | Definition | OriginalWebSite | Fisher-Pvalue | SelectionCounts | SelectionSize | Count | Size | FDR | Enrichment\_Score | GeneRatio | Genes |
| --- | --- | --- | --- | --- | --- | --- | --- | --- | --- | --- | --- |
| hsa05211 | Renal cell carcinoma - Homo sapiens (human) | http://www.genome.jp/kegg-bin/show\_pathway?hsa05211+1026+1398+1399+54583+112399+2113+2549+2885+3725+5594+4233+56924+5291+8503+5908+6654+6655+7042 | 1.285095e-08 | 18 | 424 | 69 | 7906 | 2.455643e-06 | 7.891065 | 0.042453 | CDKN1A//CRK//CRKL//EGLN1//EGLN3//ETS1//GAB1//GRB2//JUN//MAPK1//MET//PAK6//PIK3CB//PIK3R3//RAP1B//SOS1//SOS2//TGFB2 |
| hsa05200 | Pathways in cancer - Homo sapiens (human) | http://www.genome.jp/kegg-bin/show\_pathway?hsa05200+109+26060+367+10018+805+8030+894+9134+1021+1026+1398+1399+28514+1869+1871+1906+54583+112399+2099+2113+11211+8323+1647+2776+10681+2885+2939+3439+3479+3558+3655+3685+3716+3725+3909+1902+23566+4040+5594+4233+4313+5291+8503+5366+5468+5567+5728+5734+5915+9252+6500+6654+6655+6774+6775+83439+7042+7048+7423+7476 | 1.470445e-08 | 60 | 424 | 529 | 7906 | 2.455643e-06 | 7.832551 | 0.141509 | ADCY3//APPL1//AR//BCL2L11//CALM2//CCDC6//CCND2//CCNE2//CDK6//CDKN1A//CRK//CRKL//DLL1//E2F1//E2F3//EDN1//EGLN1//EGLN3//ESR1//ETS1//FZD10//FZD6//GADD45A//GNAQ//GNB5//GRB2//GSTA2//IFNA1//IGF1//IL2//ITGA6//ITGAV//JAK1//JUN//LAMA3//LPAR1//LPAR3//LRP6//MAPK1//MET//MMP2//PIK3CB//PIK3R3//PMAIP1//PPARG//PRKACB//PTEN//PTGER4//RARB//RPS6KA5//SKP1//SOS1//SOS2//STAT3//STAT4//TCF7L1//TGFB2//TGFBR2//VEGFB//WNT7A |
| hsa04151 | PI3K-Akt signaling pathway - Homo sapiens (human) | http://www.genome.jp/kegg-bin/show\_pathway?hsa04151+10018+894+9134+1021+1026+1129+1278+1942+1946+1969+2069+10681+2885+3439+3479+3558+3678+3655+3685+3696+3716+3791+3909+1902+23566+5594+4233+4602+4908+5008+5291+8503+5515+5562+5728+5934+6009+6654+6655+7248+7423+7532 | 7.951924e-07 | 42 | 424 | 354 | 7906 | 8.853142e-05 | 6.099528 | 0.099057 | BCL2L11//CCND2//CCNE2//CDK6//CDKN1A//CHRM2//COL1A2//EFNA1//EFNA5//EPHA2//EREG//GNB5//GRB2//IFNA1//IGF1//IL2//ITGA5//ITGA6//ITGAV//ITGB8//JAK1//KDR//LAMA3//LPAR1//LPAR3//MAPK1//MET//MYB//NTF3//OSM//PIK3CB//PIK3R3//PPP2CA//PRKAA1//PTEN//RBL2//RHEB//SOS1//SOS2//TSC1//VEGFB//YWHAG |
| hsa04068 | FoxO signaling pathway - Homo sapiens (human) | http://www.genome.jp/kegg-bin/show\_pathway?hsa04068+10018+664+894+901+1026+1647+2885+3479+10365+5594+5291+8503+5562+5728+5934+1901+6517+6654+6655+6774+7042+7048 | 1.475139e-06 | 22 | 424 | 131 | 7906 | 1.231741e-04 | 5.831167 | 0.051887 | BCL2L11//BNIP3//CCND2//CCNG2//CDKN1A//GADD45A//GRB2//IGF1//KLF2//MAPK1//PIK3CB//PIK3R3//PRKAA1//PTEN//RBL2//S1PR1//SLC2A4//SOS1//SOS2//STAT3//TGFB2//TGFBR2 |
| hsa00053 | Ascorbate and aldarate metabolism - Homo sapiens (human) | http://www.genome.jp/kegg-bin/show\_pathway?hsa00053+54658+54575+54659+54657+54579+54578+54577+54576+54600 | 6.652794e-06 | 9 | 424 | 27 | 7906 | 3.462363e-04 | 5.176996 | 0.021226 | UGT1A1//UGT1A10//UGT1A3//UGT1A4//UGT1A5//UGT1A6//UGT1A7//UGT1A8//UGT1A9 |
| hsa04010 | MAPK signaling pathway - Homo sapiens (human) | http://www.genome.jp/kegg-bin/show\_pathway?hsa04010+1398+1399+1843+11221+1844+1847+1942+1946+1969+2069+1647+2885+3479+3725+3791+8649+6416+10746+4217+5594+4233+2872+4908+5534+5567+5908+8986+9252+6654+6655+51347+7042+7048+7124+7423 | 6.906414e-06 | 35 | 424 | 295 | 7906 | 3.462363e-04 | 5.160747 | 0.082547 | CRK//CRKL//DUSP1//DUSP10//DUSP2//DUSP5//EFNA1//EFNA5//EPHA2//EREG//GADD45A//GRB2//IGF1//JUN//KDR//LAMTOR3//MAP2K4//MAP3K2//MAP3K5//MAPK1//MET//MKNK2//NTF3//PPP3R1//PRKACB//RAP1B//RPS6KA4//RPS6KA5//SOS1//SOS2//TAOK3//TGFB2//TGFBR2//TNF//VEGFB |
| hsa00040 | Pentose and glucuronate interconversions - Homo sapiens (human) | http://www.genome.jp/kegg-bin/show\_pathway?hsa00040+7360+54658+54575+54659+54657+54579+54578+54577+54576+54600 | 7.256450e-06 | 10 | 424 | 34 | 7906 | 3.462363e-04 | 5.139276 | 0.023585 | UGP2//UGT1A1//UGT1A10//UGT1A3//UGT1A4//UGT1A5//UGT1A6//UGT1A7//UGT1A8//UGT1A9 |
| hsa04012 | ErbB signaling pathway - Homo sapiens (human) | http://www.genome.jp/kegg-bin/show\_pathway?hsa04012+1026+1398+1399+2069+2549+2885+3725+6416+5594+4690+3084+56924+5291+8503+6654+6655 | 9.074014e-06 | 16 | 424 | 85 | 7906 | 3.513700e-04 | 5.042201 | 0.037736 | CDKN1A//CRK//CRKL//EREG//GAB1//GRB2//JUN//MAP2K4//MAPK1//NCK1//NRG1//PAK6//PIK3CB//PIK3R3//SOS1//SOS2 |
| hsa05220 | Chronic myeloid leukemia - Homo sapiens (human) | http://www.genome.jp/kegg-bin/show\_pathway?hsa05220+1021+1026+1398+1399+1869+1871+1647+2885+5594+5291+8503+6654+6655+7042+7048 | 9.468054e-06 | 15 | 424 | 76 | 7906 | 3.513700e-04 | 5.023739 | 0.035377 | CDK6//CDKN1A//CRK//CRKL//E2F1//E2F3//GADD45A//GRB2//MAPK1//PIK3CB//PIK3R3//SOS1//SOS2//TGFB2//TGFBR2 |
| hsa05210 | Colorectal cancer - Homo sapiens (human) | http://www.genome.jp/kegg-bin/show\_pathway?hsa05210+26060+10018+1026+2069+1647+2885+3725+5594+5291+8503+5366+6654+6655+83439+7042+7048 | 1.060918e-05 | 16 | 424 | 86 | 7906 | 3.543467e-04 | 4.974318 | 0.037736 | APPL1//BCL2L11//CDKN1A//EREG//GADD45A//GRB2//JUN//MAPK1//PIK3CB//PIK3R3//PMAIP1//SOS1//SOS2//TCF7L1//TGFB2//TGFBR2 |
| hsa00983 | Drug metabolism - other enzymes - Homo sapiens (human) | http://www.genome.jp/kegg-bin/show\_pathway?hsa00983+51727+2939+3251+3614+4830+6241+54658+54575+54659+54657+54579+54578+54577+54576+54600 | 1.549421e-05 | 15 | 424 | 79 | 7906 | 4.312554e-04 | 4.809831 | 0.035377 | CMPK1//GSTA2//HPRT1//IMPDH1//NME1//RRM2//UGT1A1//UGT1A10//UGT1A3//UGT1A4//UGT1A5//UGT1A6//UGT1A7//UGT1A8//UGT1A9 |
| hsa01521 | EGFR tyrosine kinase inhibitor resistance - Homo sapiens (human) | http://www.genome.jp/kegg-bin/show\_pathway?hsa01521+10018+2549+2885+3479+3716+3791+5594+4233+3084+5291+8503+5728+6654+6655+6774 | 1.549421e-05 | 15 | 424 | 79 | 7906 | 4.312554e-04 | 4.809831 | 0.035377 | BCL2L11//GAB1//GRB2//IGF1//JAK1//KDR//MAPK1//MET//NRG1//PIK3CB//PIK3R3//PTEN//SOS1//SOS2//STAT3 |
| hsa04115 | p53 signaling pathway - Homo sapiens (human) | http://www.genome.jp/kegg-bin/show\_pathway?hsa04115+894+9134+901+1021+1026+1647+3479+4194+5366+5728+6241+27244+143686+64393 | 2.243937e-05 | 14 | 424 | 72 | 7906 | 5.765191e-04 | 4.648989 | 0.033019 | CCND2//CCNE2//CCNG2//CDK6//CDKN1A//GADD45A//IGF1//MDM4//PMAIP1//PTEN//RRM2//SESN1//SESN3//ZMAT3 |
| hsa05225 | Hepatocellular carcinoma - Homo sapiens (human) | http://www.genome.jp/kegg-bin/show\_pathway?hsa05225+1021+1026+1869+1871+11211+8323+2549+1647+2885+2939+4040+5594+4233+5291+8503+5728+6603+6654+6655+83439+7042+7048+7476 | 2.891710e-05 | 23 | 424 | 168 | 7906 | 6.289293e-04 | 4.538845 | 0.054245 | CDK6//CDKN1A//E2F1//E2F3//FZD10//FZD6//GAB1//GADD45A//GRB2//GSTA2//LRP6//MAPK1//MET//PIK3CB//PIK3R3//PTEN//SMARCD2//SOS1//SOS2//TCF7L1//TGFB2//TGFBR2//WNT7A |
| hsa05205 | Proteoglycans in cancer - Homo sapiens (human) | http://www.genome.jp/kegg-bin/show\_pathway?hsa05205+1026+1278+2099+11211+8323+2549+2885+3479+3678+3685+3791+5594+4233+4313+4478+5291+8503+4659+54776+5567+6654+6655+6774+7042+7124+7476 | 3.157290e-05 | 26 | 424 | 204 | 7906 | 6.289293e-04 | 4.500686 | 0.061321 | CDKN1A//COL1A2//ESR1//FZD10//FZD6//GAB1//GRB2//IGF1//ITGA5//ITGAV//KDR//MAPK1//MET//MMP2//MSN//PIK3CB//PIK3R3//PPP1R12A//PPP1R12C//PRKACB//SOS1//SOS2//STAT3//TGFB2//TNF//WNT7A |
| hsa04360 | Axon guidance - Homo sapiens (human) | http://www.genome.jp/kegg-bin/show\_pathway?hsa04360+659+1073+1808+1942+1946+55740+1969+2043+2044+2045+5594+4233+4690+59277+22854+56924+5291+8503+5534+56963+5998+10509+9353+7222 | 3.320556e-05 | 24 | 424 | 181 | 7906 | 6.289293e-04 | 4.478789 | 0.056604 | BMPR2//CFL2//DPYSL2//EFNA1//EFNA5//ENAH//EPHA2//EPHA4//EPHA5//EPHA7//MAPK1//MET//NCK1//NTN4//NTNG1//PAK6//PIK3CB//PIK3R3//PPP3R1//RGMA//RGS3//SEMA4B//SLIT2//TRPC3 |
| hsa05224 | Breast cancer - Homo sapiens (human) | http://www.genome.jp/kegg-bin/show\_pathway?hsa05224+1021+1026+28514+1869+1871+2099+11211+8323+1647+2885+3479+3725+4040+5594+5291+8503+5728+6654+6655+83439+7476 | 3.381032e-05 | 21 | 424 | 147 | 7906 | 6.289293e-04 | 4.470951 | 0.049528 | CDK6//CDKN1A//DLL1//E2F1//E2F3//ESR1//FZD10//FZD6//GADD45A//GRB2//IGF1//JUN//LRP6//MAPK1//PIK3CB//PIK3R3//PTEN//SOS1//SOS2//TCF7L1//WNT7A |
| hsa04350 | TGF-beta signaling pathway - Homo sapiens (human) | http://www.genome.jp/kegg-bin/show\_pathway?hsa04350+90+25805+659+1875+3625+5594+9241+5515+56963+285704+6500+7042+7048+7050+7124+9372 | 3.389440e-05 | 16 | 424 | 94 | 7906 | 6.289293e-04 | 4.469872 | 0.037736 | ACVR1//BAMBI//BMPR2//E2F5//INHBB//MAPK1//NOG//PPP2CA//RGMA//RGMB//SKP1//TGFB2//TGFBR2//TGIF1//TNF//ZFYVE9 |
| hsa05214 | Glioma - Homo sapiens (human) | http://www.genome.jp/kegg-bin/show\_pathway?hsa05214+805+1021+1026+1869+1871+1647+2885+3479+5594+5291+8503+5728+6654+6655 | 3.632015e-05 | 14 | 424 | 75 | 7906 | 6.302386e-04 | 4.439852 | 0.033019 | CALM2//CDK6//CDKN1A//E2F1//E2F3//GADD45A//GRB2//IGF1//MAPK1//PIK3CB//PIK3R3//PTEN//SOS1//SOS2 |
| hsa05223 | Non-small cell lung cancer - Homo sapiens (human) | http://www.genome.jp/kegg-bin/show\_pathway?hsa05223+1021+1026+1869+1871+1647+2885+5594+5291+8503+5915+6654+6655+6774 | 3.811760e-05 | 13 | 424 | 66 | 7906 | 6.302386e-04 | 4.418874 | 0.030660 | CDK6//CDKN1A//E2F1//E2F3//GADD45A//GRB2//MAPK1//PIK3CB//PIK3R3//RARB//SOS1//SOS2//STAT3 |
| hsa04218 | Cellular senescence - Homo sapiens (human) | http://www.genome.jp/kegg-bin/show\_pathway?hsa04218+805+894+9134+1021+1026+1869+1871+1875+2113+1647+10114+5594+5291+8503+5534+5728+5934+6009+8878+7042+7048+7248 | 4.026242e-05 | 22 | 424 | 160 | 7906 | 6.302386e-04 | 4.395100 | 0.051887 | CALM2//CCND2//CCNE2//CDK6//CDKN1A//E2F1//E2F3//E2F5//ETS1//GADD45A//HIPK3//MAPK1//PIK3CB//PIK3R3//PPP3R1//PTEN//RBL2//RHEB//SQSTM1//TGFB2//TGFBR2//TSC1 |
| hsa05226 | Gastric cancer - Homo sapiens (human) | http://www.genome.jp/kegg-bin/show\_pathway?hsa05226+9134+1026+1869+1871+11211+8323+2549+1647+2885+4040+5594+4233+5291+8503+5915+6654+6655+83439+7042+7048+7476 | 4.151272e-05 | 21 | 424 | 149 | 7906 | 6.302386e-04 | 4.381819 | 0.049528 | CCNE2//CDKN1A//E2F1//E2F3//FZD10//FZD6//GAB1//GADD45A//GRB2//LRP6//MAPK1//MET//PIK3CB//PIK3R3//RARB//SOS1//SOS2//TCF7L1//TGFB2//TGFBR2//WNT7A |
| hsa05418 | Fluid shear stress and atherosclerosis - Homo sapiens (human) | http://www.genome.jp/kegg-bin/show\_pathway?hsa05418+90+659+805+6347+1843+1906+2939+3685+3725+3791+10365+6416+4217+4313+51588+5291+8503+5562+8878+7124 | 4.676292e-05 | 20 | 424 | 139 | 7906 | 6.746025e-04 | 4.330098 | 0.047170 | ACVR1//BMPR2//CALM2//CCL2//DUSP1//EDN1//GSTA2//ITGAV//JUN//KDR//KLF2//MAP2K4//MAP3K5//MMP2//PIAS4//PIK3CB//PIK3R3//PRKAA1//SQSTM1//TNF |
| hsa05206 | MicroRNAs in cancer - Homo sapiens (human) | http://www.genome.jp/kegg-bin/show\_pathway?hsa05206+10018+659+894+9134+1021+1026+1398+1399+1789+1869+1871+1942+1946+2885+8091+3678+9493+5594+4082+4194+4233+5291+8503+5728+8434+9252+6654+6655+6659+6774+7042+7329+6935+23414 | 4.927287e-05 | 34 | 424 | 310 | 7906 | 6.746025e-04 | 4.307392 | 0.080189 | BCL2L11//BMPR2//CCND2//CCNE2//CDK6//CDKN1A//CRK//CRKL//DNMT3B//E2F1//E2F3//EFNA1//EFNA5//GRB2//HMGA2//ITGA5//KIF23//MAPK1//MARCKS//MDM4//MET//PIK3CB//PIK3R3//PTEN//RECK//RPS6KA5//SOS1//SOS2//SOX4//STAT3//TGFB2//UBE2I//ZEB1//ZFPM2 |
| hsa05215 | Prostate cancer - Homo sapiens (human) | http://www.genome.jp/kegg-bin/show\_pathway?hsa05215+367+9134+1026+1869+1871+2078+2885+3479+5594+5291+8503+5728+6654+6655+83439+6935 | 5.049420e-05 | 16 | 424 | 97 | 7906 | 6.746025e-04 | 4.296758 | 0.037736 | AR//CCNE2//CDKN1A//E2F1//E2F3//ERG//GRB2//IGF1//MAPK1//PIK3CB//PIK3R3//PTEN//SOS1//SOS2//TCF7L1//ZEB1 |
| hsa04510 | Focal adhesion - Homo sapiens (human) | http://www.genome.jp/kegg-bin/show\_pathway?hsa04510+894+1278+1398+1399+2885+3479+3678+3655+3685+3696+3725+3791+3909+5594+4233+56924+5291+8503+4659+54776+5728+5908+6654+6655+7423 | 5.705941e-05 | 25 | 424 | 199 | 7906 | 7.104935e-04 | 4.243673 | 0.058962 | CCND2//COL1A2//CRK//CRKL//GRB2//IGF1//ITGA5//ITGA6//ITGAV//ITGB8//JUN//KDR//LAMA3//MAPK1//MET//PAK6//PIK3CB//PIK3R3//PPP1R12A//PPP1R12C//PTEN//RAP1B//SOS1//SOS2//VEGFB |
| hsa01522 | Endocrine resistance - Homo sapiens (human) | http://www.genome.jp/kegg-bin/show\_pathway?hsa01522+109+1026+28514+1869+1871+2099+2885+3479+3725+5594+4313+5291+8503+5567+6654+6655 | 5.743510e-05 | 16 | 424 | 98 | 7906 | 7.104935e-04 | 4.240823 | 0.037736 | ADCY3//CDKN1A//DLL1//E2F1//E2F3//ESR1//GRB2//IGF1//JUN//MAPK1//MMP2//PIK3CB//PIK3R3//PRKACB//SOS1//SOS2 |
| hsa04150 | mTOR signaling pathway - Homo sapiens (human) | http://www.genome.jp/kegg-bin/show\_pathway?hsa04150+6249+96459+11211+8323+2885+3479+8649+4040+5594+5291+8503+5562+5728+6009+58528+6654+6655+7124+7248+9706+7476 | 6.177642e-05 | 21 | 424 | 153 | 7906 | 7.369045e-04 | 4.209177 | 0.049528 | CLIP1//FNIP1//FZD10//FZD6//GRB2//IGF1//LAMTOR3//LRP6//MAPK1//PIK3CB//PIK3R3//PRKAA1//PTEN//RHEB//RRAGD//SOS1//SOS2//TNF//TSC1//ULK2//WNT7A |
| hsa05163 | Human cytomegalovirus infection - Homo sapiens (human) | http://www.genome.jp/kegg-bin/show\_pathway?hsa05163+109+805+6347+1021+1026+1398+1399+1869+1871+2776+10681+2885+3439+3685+3716+5594+5291+8503+5534+5567+5734+6009+6654+6655+6774+7124+7248 | 6.508042e-05 | 27 | 424 | 225 | 7906 | 7.495469e-04 | 4.186550 | 0.063679 | ADCY3//CALM2//CCL2//CDK6//CDKN1A//CRK//CRKL//E2F1//E2F3//GNAQ//GNB5//GRB2//IFNA1//ITGAV//JAK1//MAPK1//PIK3CB//PIK3R3//PPP3R1//PRKACB//PTGER4//RHEB//SOS1//SOS2//STAT3//TNF//TSC1 |
| hsa04550 | Signaling pathways regulating pluripotency of stem cells - Homo sapiens (human) | http://www.genome.jp/kegg-bin/show\_pathway?hsa04550+90+659+11211+8323+2885+9421+3479+3625+3716+3720+9314+5594+4762+84333+5291+8503+5978+6774+7476 | 1.600986e-04 | 19 | 424 | 140 | 7906 | 1.782431e-03 | 3.795612 | 0.044811 | ACVR1//BMPR2//FZD10//FZD6//GRB2//HAND1//IGF1//INHBB//JAK1//JARID2//KLF4//MAPK1//NEUROG1//PCGF5//PIK3CB//PIK3R3//REST//STAT3//WNT7A |
| hsa04710 | Circadian rhythm - Homo sapiens (human) | http://www.genome.jp/kegg-bin/show\_pathway?hsa04710+8553+79365+9575+1408+4862+8864+5562+6500 | 1.693963e-04 | 8 | 424 | 31 | 7906 | 1.803082e-03 | 3.771096 | 0.018868 | BHLHE40//BHLHE41//CLOCK//CRY2//NPAS2//PER2//PRKAA1//SKP1 |
| hsa04810 | Regulation of actin cytoskeleton - Homo sapiens (human) | http://www.genome.jp/kegg-bin/show\_pathway?hsa04810+50649+1073+1129+1133+1398+1399+55740+10788+3678+3655+3685+3696+1902+5594+4478+56924+5217+5291+8503+200576+4659+54776+6654+6655+8976 | 1.727504e-04 | 25 | 424 | 213 | 7906 | 1.803082e-03 | 3.762581 | 0.058962 | ARHGEF4//CFL2//CHRM2//CHRM5//CRK//CRKL//ENAH//IQGAP2//ITGA5//ITGA6//ITGAV//ITGB8//LPAR1//MAPK1//MSN//PAK6//PFN2//PIK3CB//PIK3R3//PIKFYVE//PPP1R12A//PPP1R12C//SOS1//SOS2//WASL |
| hsa03018 | RNA degradation - Homo sapiens (human) | http://www.genome.jp/kegg-bin/show\_pathway?hsa03018+694+7832+10950+57472+246175+29883+167227+1656+140886+255967+5214+10140+10766 | 2.603228e-04 | 13 | 424 | 79 | 7906 | 2.634782e-03 | 3.584488 | 0.030660 | BTG1//BTG2//BTG3//CNOT6//CNOT6L//CNOT7//DCP2//DDX6//PABPC5//PAN3//PFKP//TOB1//TOB2 |
| hsa00860 | Porphyrin and chlorophyll metabolism - Homo sapiens (human) | http://www.genome.jp/kegg-bin/show\_pathway?hsa00860+54658+54575+54659+54657+54579+54578+54577+54576+54600 | 3.085150e-04 | 9 | 424 | 42 | 7906 | 3.030706e-03 | 3.510724 | 0.021226 | UGT1A1//UGT1A10//UGT1A3//UGT1A4//UGT1A5//UGT1A6//UGT1A7//UGT1A8//UGT1A9 |
| hsa05165 | Human papillomavirus infection - Homo sapiens (human) | http://www.genome.jp/kegg-bin/show\_pathway?hsa05165+894+9134+1021+1026+1278+1869+11211+8323+2885+3439+3659+10379+3678+3655+3685+3696+3716+3909+5594+5291+8503+5515+5567+5728+5734+5934+6009+6654+6655+83439+7124+7248+7476 | 3.688104e-04 | 33 | 424 | 330 | 7906 | 3.519505e-03 | 3.433197 | 0.077830 | CCND2//CCNE2//CDK6//CDKN1A//COL1A2//E2F1//FZD10//FZD6//GRB2//IFNA1//IRF1//IRF9//ITGA5//ITGA6//ITGAV//ITGB8//JAK1//LAMA3//MAPK1//PIK3CB//PIK3R3//PPP2CA//PRKACB//PTEN//PTGER4//RBL2//RHEB//SOS1//SOS2//TCF7L1//TNF//TSC1//WNT7A |
| hsa05161 | Hepatitis B - Homo sapiens (human) | http://www.genome.jp/kegg-bin/show\_pathway?hsa05161+9134+1026+1869+1871+1959+2885+3439+3716+3725+6416+5594+5291+8503+6654+6655+6774+6775+7042+7048+7124 | 3.940791e-04 | 20 | 424 | 162 | 7906 | 3.656178e-03 | 3.404417 | 0.047170 | CCNE2//CDKN1A//E2F1//E2F3//EGR2//GRB2//IFNA1//JAK1//JUN//MAP2K4//MAPK1//PIK3CB//PIK3R3//SOS1//SOS2//STAT3//STAT4//TGFB2//TGFBR2//TNF |
| hsa04114 | Oocyte meiosis - Homo sapiens (human) | http://www.genome.jp/kegg-bin/show\_pathway?hsa04114+109+367+805+9134+8697+64506+132864+22849+80315+3479+5594+5515+5534+5567+6500+9748+7532 | 4.544807e-04 | 17 | 424 | 128 | 7906 | 4.102609e-03 | 3.342485 | 0.040094 | ADCY3//AR//CALM2//CCNE2//CDC23//CPEB1//CPEB2//CPEB3//CPEB4//IGF1//MAPK1//PPP2CA//PPP3R1//PRKACB//SKP1//SLK//YWHAG |
| hsa05212 | Pancreatic cancer - Homo sapiens (human) | http://www.genome.jp/kegg-bin/show\_pathway?hsa05212+1021+1026+1869+1871+1647+3716+5594+5291+8503+6774+7042+7048 | 5.811870e-04 | 12 | 424 | 75 | 7906 | 5.108328e-03 | 3.235684 | 0.028302 | CDK6//CDKN1A//E2F1//E2F3//GADD45A//JAK1//MAPK1//PIK3CB//PIK3R3//STAT3//TGFB2//TGFBR2 |
| hsa05160 | Hepatitis C - Homo sapiens (human) | http://www.genome.jp/kegg-bin/show\_pathway?hsa05160+1021+1026+5010+1869+1871+2885+3439+10379+3716+3949+5594+5291+8503+5515+6654+6655+6774+7124+7532 | 5.987043e-04 | 19 | 424 | 155 | 7906 | 5.127365e-03 | 3.222788 | 0.044811 | CDK6//CDKN1A//CLDN11//E2F1//E2F3//GRB2//IFNA1//IRF9//JAK1//LDLR//MAPK1//PIK3CB//PIK3R3//PPP2CA//SOS1//SOS2//STAT3//TNF//YWHAG |
| hsa05166 | Human T-cell leukemia virus 1 infection - Homo sapiens (human) | http://www.genome.jp/kegg-bin/show\_pathway?hsa05166+109+894+9134+8697+1026+1869+1871+1959+2113+8061+3558+3716+3725+8850+6416+5594+5291+8503+5534+5567+5728+7042+7048+7124 | 6.435959e-04 | 24 | 424 | 219 | 7906 | 5.374026e-03 | 3.191387 | 0.056604 | ADCY3//CCND2//CCNE2//CDC23//CDKN1A//E2F1//E2F3//EGR2//ETS1//FOSL1//IL2//JAK1//JUN//KAT2B//MAP2K4//MAPK1//PIK3CB//PIK3R3//PPP3R1//PRKACB//PTEN//TGFB2//TGFBR2//TNF |
| hsa05231 | Choline metabolism in cancer - Homo sapiens (human) | http://www.genome.jp/kegg-bin/show\_pathway?hsa05231+8526+160851+2885+3725+10434+5594+5291+8503+6009+23446+6654+6655+7248+10810 | 6.835417e-04 | 14 | 424 | 98 | 7906 | 5.568364e-03 | 3.165235 | 0.033019 | DGKE//DGKH//GRB2//JUN//LYPLA1//MAPK1//PIK3CB//PIK3R3//RHEB//SLC44A1//SOS1//SOS2//TSC1//WASF3 |
| hsa04933 | AGE-RAGE signaling pathway in diabetic complications - Homo sapiens (human) | http://www.genome.jp/kegg-bin/show\_pathway?hsa04933+6347+1278+1906+2152+3725+5594+4313+5291+8503+6774+7042+7048+7124+7423 | 8.394433e-04 | 14 | 424 | 100 | 7906 | 6.612620e-03 | 3.076009 | 0.033019 | CCL2//COL1A2//EDN1//F3//JUN//MAPK1//MMP2//PIK3CB//PIK3R3//STAT3//TGFB2//TGFBR2//TNF//VEGFB |
| hsa04211 | Longevity regulating pathway - Homo sapiens (human) | http://www.genome.jp/kegg-bin/show\_pathway?hsa04211+109+79602+26060+3479+5291+8503+5468+5562+5567+6009+27244+143686+7248 | 8.513253e-04 | 13 | 424 | 89 | 7906 | 6.612620e-03 | 3.069904 | 0.030660 | ADCY3//ADIPOR2//APPL1//IGF1//PIK3CB//PIK3R3//PPARG//PRKAA1//PRKACB//RHEB//SESN1//SESN3//TSC1 |
| hsa05213 | Endometrial cancer - Homo sapiens (human) | http://www.genome.jp/kegg-bin/show\_pathway?hsa05213+1026+1647+2885+5594+5291+8503+5728+6654+6655+83439 | 9.082757e-04 | 10 | 424 | 58 | 7906 | 6.894638e-03 | 3.041782 | 0.023585 | CDKN1A//GADD45A//GRB2//MAPK1//PIK3CB//PIK3R3//PTEN//SOS1//SOS2//TCF7L1 |
| hsa04910 | Insulin signaling pathway - Homo sapiens (human) | http://www.genome.jp/kegg-bin/show\_pathway?hsa04910+805+1398+1399+2885+5594+2872+5291+8503+5562+5567+5577+6009+6517+6654+6655+9322+7248 | 9.986674e-04 | 17 | 424 | 137 | 7906 | 7.412331e-03 | 3.000579 | 0.040094 | CALM2//CRK//CRKL//GRB2//MAPK1//MKNK2//PIK3CB//PIK3R3//PRKAA1//PRKACB//PRKAR2B//RHEB//SLC2A4//SOS1//SOS2//TRIP10//TSC1 |
| hsa04917 | Prolactin signaling pathway - Homo sapiens (human) | http://www.genome.jp/kegg-bin/show\_pathway?hsa04917+894+2099+2885+3659+5594+5291+8503+9655+6654+6655+6774 | 1.139302e-03 | 11 | 424 | 70 | 7906 | 8.272326e-03 | 2.943361 | 0.025943 | CCND2//ESR1//GRB2//IRF1//MAPK1//PIK3CB//PIK3R3//SOCS5//SOS1//SOS2//STAT3 |
| hsa05222 | Small cell lung cancer - Homo sapiens (human) | http://www.genome.jp/kegg-bin/show\_pathway?hsa05222+9134+1021+1026+1869+1871+1647+3655+3685+3909+5291+8503+5728+5915 | 1.168182e-03 | 13 | 424 | 92 | 7906 | 8.301548e-03 | 2.932490 | 0.030660 | CCNE2//CDK6//CDKN1A//E2F1//E2F3//GADD45A//ITGA6//ITGAV//LAMA3//PIK3CB//PIK3R3//PTEN//RARB |
| hsa04926 | Relaxin signaling pathway - Homo sapiens (human) | http://www.genome.jp/kegg-bin/show\_pathway?hsa04926+109+1278+1906+10681+2885+3725+6416+5594+4313+5291+8503+5567+6654+6655+7048+7423 | 1.400670e-03 | 16 | 424 | 129 | 7906 | 9.746329e-03 | 2.853664 | 0.037736 | ADCY3//COL1A2//EDN1//GNB5//GRB2//JUN//MAP2K4//MAPK1//MMP2//PIK3CB//PIK3R3//PRKACB//SOS1//SOS2//TGFBR2//VEGFB |
| hsa05218 | Melanoma - Homo sapiens (human) | http://www.genome.jp/kegg-bin/show\_pathway?hsa05218+1021+1026+1869+1871+1647+3479+5594+4233+5291+8503+5728 | 1.445123e-03 | 11 | 424 | 72 | 7906 | 9.850434e-03 | 2.840095 | 0.025943 | CDK6//CDKN1A//E2F1//E2F3//GADD45A//IGF1//MAPK1//MET//PIK3CB//PIK3R3//PTEN |
| hsa04722 | Neurotrophin signaling pathway - Homo sapiens (human) | http://www.genome.jp/kegg-bin/show\_pathway?hsa04722+805+1398+1399+2549+2885+3725+4217+5594+4908+5291+8503+5908+9252+6654+6655 | 1.661988e-03 | 15 | 424 | 119 | 7906 | 1.110208e-02 | 2.779372 | 0.035377 | CALM2//CRK//CRKL//GAB1//GRB2//JUN//MAP3K5//MAPK1//NTF3//PIK3CB//PIK3R3//RAP1B//RPS6KA5//SOS1//SOS2 |
| hsa04015 | Rap1 signaling pathway - Homo sapiens (human) | http://www.genome.jp/kegg-bin/show\_pathway?hsa04015+109+805+1398+1399+1942+1946+55740+1969+2776+3479+3791+1902+23566+5594+4233+5028+5217+5291+8503+5908+11069+7423 | 1.923929e-03 | 22 | 424 | 210 | 7906 | 1.259985e-02 | 2.715811 | 0.051887 | ADCY3//CALM2//CRK//CRKL//EFNA1//EFNA5//ENAH//EPHA2//GNAQ//IGF1//KDR//LPAR1//LPAR3//MAPK1//MET//P2RY1//PFN2//PIK3CB//PIK3R3//RAP1B//RAPGEF4//VEGFB |
| hsa04540 | Gap junction - Homo sapiens (human) | http://www.genome.jp/kegg-bin/show\_pathway?hsa04540+109+153+1812+2697+2776+2885+1902+10746+5594+5567+6654+6655 | 2.434089e-03 | 12 | 424 | 88 | 7906 | 1.563434e-02 | 2.613664 | 0.028302 | ADCY3//ADRB1//DRD1//GJA1//GNAQ//GRB2//LPAR1//MAP3K2//MAPK1//PRKACB//SOS1//SOS2 |
| hsa04110 | Cell cycle - Homo sapiens (human) | http://www.genome.jp/kegg-bin/show\_pathway?hsa04110+894+9134+8556+8697+1021+1026+1869+1871+1875+1647+5885+5934+6500+7042+7532 | 2.507822e-03 | 15 | 424 | 124 | 7906 | 1.580401e-02 | 2.600703 | 0.035377 | CCND2//CCNE2//CDC14A//CDC23//CDK6//CDKN1A//E2F1//E2F3//E2F5//GADD45A//RAD21//RBL2//SKP1//TGFB2//YWHAG |
| hsa00830 | Retinol metabolism - Homo sapiens (human) | http://www.genome.jp/kegg-bin/show\_pathway?hsa00830+157506+54658+54575+54659+54657+54579+54578+54577+54576+54600 | 2.807222e-03 | 10 | 424 | 67 | 7906 | 1.736319e-02 | 2.551723 | 0.023585 | RDH10//UGT1A1//UGT1A10//UGT1A3//UGT1A4//UGT1A5//UGT1A6//UGT1A7//UGT1A8//UGT1A9 |
| hsa04380 | Osteoclast differentiation - Homo sapiens (human) | http://www.genome.jp/kegg-bin/show\_pathway?hsa04380+2274+8061+2885+10379+3716+3725+5594+5291+8503+5468+5534+8878+7042+7048+7124 | 3.417308e-03 | 15 | 424 | 128 | 7906 | 2.075238e-02 | 2.466316 | 0.035377 | FHL2//FOSL1//GRB2//IRF9//JAK1//JUN//MAPK1//PIK3CB//PIK3R3//PPARG//PPP3R1//SQSTM1//TGFB2//TGFBR2//TNF |
| hsa04934 | Cushing syndrome - Homo sapiens (human) | http://www.genome.jp/kegg-bin/show\_pathway?hsa04934+109+9134+1021+1026+1869+1871+11211+8323+2776+3739+3949+5594+5567+5908+51655+83439+7476 | 3.805202e-03 | 17 | 424 | 155 | 7906 | 2.269531e-02 | 2.419622 | 0.040094 | ADCY3//CCNE2//CDK6//CDKN1A//E2F1//E2F3//FZD10//FZD6//GNAQ//KCNA4//LDLR//MAPK1//PRKACB//RAP1B//RASD1//TCF7L1//WNT7A |
| hsa04912 | GnRH signaling pathway - Homo sapiens (human) | http://www.genome.jp/kegg-bin/show\_pathway?hsa04912+109+805+2776+2885+3725+6416+10746+5594+4313+5567+6654+6655 | 3.878712e-03 | 12 | 424 | 93 | 7906 | 2.272789e-02 | 2.411313 | 0.028302 | ADCY3//CALM2//GNAQ//GRB2//JUN//MAP2K4//MAP3K2//MAPK1//MMP2//PRKACB//SOS1//SOS2 |
| hsa00140 | Steroid hormone biosynthesis - Homo sapiens (human) | http://www.genome.jp/kegg-bin/show\_pathway?hsa00140+54658+54575+54659+54657+54579+54578+54577+54576+54600 | 4.352795e-03 | 9 | 424 | 60 | 7906 | 2.506609e-02 | 2.361232 | 0.021226 | UGT1A1//UGT1A10//UGT1A3//UGT1A4//UGT1A5//UGT1A6//UGT1A7//UGT1A8//UGT1A9 |
| hsa00982 | Drug metabolism - cytochrome P450 - Homo sapiens (human) | http://www.genome.jp/kegg-bin/show\_pathway?hsa00982+2939+54658+54575+54659+54657+54579+54578+54577+54576+54600 | 4.783445e-03 | 10 | 424 | 72 | 7906 | 2.669901e-02 | 2.320259 | 0.023585 | GSTA2//UGT1A1//UGT1A10//UGT1A3//UGT1A4//UGT1A5//UGT1A6//UGT1A7//UGT1A8//UGT1A9 |
| hsa05135 | Yersinia infection - Homo sapiens (human) | http://www.genome.jp/kegg-bin/show\_pathway?hsa05135+64283+6347+1398+1399+9815+3558+3678+3725+6416+5594+5291+8503+7124+8976 | 4.796229e-03 | 14 | 424 | 120 | 7906 | 2.669901e-02 | 2.319100 | 0.033019 | ARHGEF28//CCL2//CRK//CRKL//GIT2//IL2//ITGA5//JUN//MAP2K4//MAPK1//PIK3CB//PIK3R3//TNF//WASL |
| hsa04072 | Phospholipase D signaling pathway - Homo sapiens (human) | http://www.genome.jp/kegg-bin/show\_pathway?hsa04072+109+8526+160851+2549+2885+1902+23566+5594+5291+8503+11069+6009+6654+6655+56848+7248 | 5.659121e-03 | 16 | 424 | 148 | 7906 | 3.098601e-02 | 2.247251 | 0.037736 | ADCY3//DGKE//DGKH//GAB1//GRB2//LPAR1//LPAR3//MAPK1//PIK3CB//PIK3R3//RAPGEF4//RHEB//SOS1//SOS2//SPHK2//TSC1 |
| hsa04014 | Ras signaling pathway - Homo sapiens (human) | http://www.genome.jp/kegg-bin/show\_pathway?hsa04014+805+1942+1946+1969+2113+2549+10681+2885+3479+3791+5594+4233+4908+56924+5291+8503+5567+5908+23179+6654+6655+7423 | 6.470280e-03 | 22 | 424 | 232 | 7906 | 3.430682e-02 | 2.189077 | 0.051887 | CALM2//EFNA1//EFNA5//EPHA2//ETS1//GAB1//GNB5//GRB2//IGF1//KDR//MAPK1//MET//NTF3//PAK6//PIK3CB//PIK3R3//PRKACB//RAP1B//RGL1//SOS1//SOS2//VEGFB |
| hsa04140 | Autophagy - animal - Homo sapiens (human) | http://www.genome.jp/kegg-bin/show\_pathway?hsa04140+55054+664+5594+29982+5291+8503+5515+5562+5567+5728+6009+58528+8878+7248+9706 | 6.471047e-03 | 15 | 424 | 137 | 7906 | 3.430682e-02 | 2.189025 | 0.035377 | ATG16L1//BNIP3//MAPK1//NRBF2//PIK3CB//PIK3R3//PPP2CA//PRKAA1//PRKACB//PTEN//RHEB//RRAGD//SQSTM1//TSC1//ULK2 |
| hsa04668 | TNF signaling pathway - Homo sapiens (human) | http://www.genome.jp/kegg-bin/show\_pathway?hsa04668+6347+1051+1906+3659+3725+6416+4217+5594+5291+8503+8986+9252+7124 | 6.739141e-03 | 13 | 424 | 112 | 7906 | 3.510220e-02 | 2.171395 | 0.030660 | CCL2//CEBPB//EDN1//IRF1//JUN//MAP2K4//MAP3K5//MAPK1//PIK3CB//PIK3R3//RPS6KA4//RPS6KA5//TNF |
| hsa04915 | Estrogen signaling pathway - Homo sapiens (human) | http://www.genome.jp/kegg-bin/show\_pathway?hsa04915+109+805+2099+2776+2885+3725+3760+342574+5594+4313+5291+8503+5567+6654+6655 | 6.915132e-03 | 15 | 424 | 138 | 7906 | 3.510220e-02 | 2.160200 | 0.035377 | ADCY3//CALM2//ESR1//GNAQ//GRB2//JUN//KCNJ3//KRT27//MAPK1//MMP2//PIK3CB//PIK3R3//PRKACB//SOS1//SOS2 |
| hsa00604 | Glycosphingolipid biosynthesis - ganglio series - Homo sapiens (human) | http://www.genome.jp/kegg-bin/show\_pathway?hsa00604+8869+256435+30815+29906 | 6.938552e-03 | 4 | 424 | 15 | 7906 | 3.510220e-02 | 2.158731 | 0.009434 | ST3GAL5//ST6GALNAC3//ST6GALNAC6//ST8SIA5 |
| hsa00980 | Metabolism of xenobiotics by cytochrome P450 - Homo sapiens (human) | http://www.genome.jp/kegg-bin/show\_pathway?hsa00980+2939+54658+54575+54659+54657+54579+54578+54577+54576+54600 | 7.041460e-03 | 10 | 424 | 76 | 7906 | 3.510220e-02 | 2.152337 | 0.023585 | GSTA2//UGT1A1//UGT1A10//UGT1A3//UGT1A4//UGT1A5//UGT1A6//UGT1A7//UGT1A8//UGT1A9 |
| hsa00534 | Glycosaminoglycan biosynthesis - heparan sulfate / heparin - Homo sapiens (human) | http://www.genome.jp/kegg-bin/show\_pathway?hsa00534+9653+9957+9955+222537+64132 | 7.892304e-03 | 5 | 424 | 24 | 7906 | 3.876514e-02 | 2.102796 | 0.011792 | HS2ST1//HS3ST1//HS3ST3A1//HS3ST5//XYLT2 |
| hsa05142 | Chagas disease (American trypanosomiasis) - Homo sapiens (human) | http://www.genome.jp/kegg-bin/show\_pathway?hsa05142+6347+2776+3558+3725+6416+5594+5291+8503+5515+7042+7048+7124 | 8.157711e-03 | 12 | 424 | 102 | 7906 | 3.919971e-02 | 2.088432 | 0.028302 | CCL2//GNAQ//IL2//JUN//MAP2K4//MAPK1//PIK3CB//PIK3R3//PPP2CA//TGFB2//TGFBR2//TNF |
| hsa05221 | Acute myeloid leukemia - Homo sapiens (human) | http://www.genome.jp/kegg-bin/show\_pathway?hsa05221+2885+5594+8864+5291+8503+6654+6655+6774+83439 | 8.215507e-03 | 9 | 424 | 66 | 7906 | 3.919971e-02 | 2.085366 | 0.021226 | GRB2//MAPK1//PER2//PIK3CB//PIK3R3//SOS1//SOS2//STAT3//TCF7L1 |
| hsa04660 | T cell receptor signaling pathway - Homo sapiens (human) | http://www.genome.jp/kegg-bin/show\_pathway?hsa04660+2885+3558+3725+5594+4690+56924+5291+8503+5534+6654+6655+7124 | 8.800657e-03 | 12 | 424 | 103 | 7906 | 4.140028e-02 | 2.055485 | 0.028302 | GRB2//IL2//JUN//MAPK1//NCK1//PAK6//PIK3CB//PIK3R3//PPP3R1//SOS1//SOS2//TNF |
| hsa05202 | Transcriptional misregulation in cancer - Homo sapiens (human) | http://www.genome.jp/kegg-bin/show\_pathway?hsa05202+64919+894+1026+1051+2078+2313+1647+8091+3479+51274+4094+4233+8013+5090+8864+5468+7048+6935 | 1.075264e-02 | 18 | 424 | 186 | 7906 | 4.988029e-02 | 1.968485 | 0.042453 | BCL11B//CCND2//CDKN1A//CEBPB//ERG//FLI1//GADD45A//HMGA2//IGF1//KLF3//MAF//MET//NR4A3//PBX3//PER2//PPARG//TGFBR2//ZEB1 |
| hsa05203 | Viral carcinogenesis - Homo sapiens (human) | http://www.genome.jp/kegg-bin/show\_pathway?hsa05203+894+9134+1021+1026+1959+2885+2960+10379+3716+3725+8850+5594+5291+8503+5366+5567+5934+6774+7532 | 1.132134e-02 | 19 | 424 | 201 | 7906 | 5.179902e-02 | 1.946102 | 0.044811 | CCND2//CCNE2//CDK6//CDKN1A//EGR2//GRB2//GTF2E1//IRF9//JAK1//JUN//KAT2B//MAPK1//PIK3CB//PIK3R3//PMAIP1//PRKACB//RBL2//STAT3//YWHAG |
| hsa04310 | Wnt signaling pathway - Homo sapiens (human) | http://www.genome.jp/kegg-bin/show\_pathway?hsa04310+25805+894+1501+8061+11211+8323+3725+4040+5534+5567+6500+64321+79718+83439+81839+7476 | 1.170805e-02 | 16 | 424 | 160 | 7906 | 5.284443e-02 | 1.931516 | 0.037736 | BAMBI//CCND2//CTNND2//FOSL1//FZD10//FZD6//JUN//LRP6//PPP3R1//PRKACB//SKP1//SOX17//TBL1XR1//TCF7L1//VANGL1//WNT7A |
| hsa05204 | Chemical carcinogenesis - Homo sapiens (human) | http://www.genome.jp/kegg-bin/show\_pathway?hsa05204+2939+54658+54575+54659+54657+54579+54578+54577+54576+54600 | 1.187742e-02 | 10 | 424 | 82 | 7906 | 5.289412e-02 | 1.925278 | 0.023585 | GSTA2//UGT1A1//UGT1A10//UGT1A3//UGT1A4//UGT1A5//UGT1A6//UGT1A7//UGT1A8//UGT1A9 |
| hsa05216 | Thyroid cancer - Homo sapiens (human) | http://www.genome.jp/kegg-bin/show\_pathway?hsa05216+8030+1026+1647+5594+5468+83439 | 1.296297e-02 | 6 | 424 | 37 | 7906 | 5.625830e-02 | 1.887295 | 0.014151 | CCDC6//CDKN1A//GADD45A//MAPK1//PPARG//TCF7L1 |
| hsa04630 | JAK-STAT signaling pathway - Homo sapiens (human) | http://www.genome.jp/kegg-bin/show\_pathway?hsa04630+894+1026+2885+3439+3558+10379+3716+5008+51588+5291+8503+9655+6654+6655+6774+6775 | 1.308608e-02 | 16 | 424 | 162 | 7906 | 5.625830e-02 | 1.883190 | 0.037736 | CCND2//CDKN1A//GRB2//IFNA1//IL2//IRF9//JAK1//OSM//PIAS4//PIK3CB//PIK3R3//SOCS5//SOS1//SOS2//STAT3//STAT4 |
| hsa01040 | Biosynthesis of unsaturated fatty acids - Homo sapiens (human) | http://www.genome.jp/kegg-bin/show\_pathway?hsa01040+11332+51+6785+79993+79966 | 1.313816e-02 | 5 | 424 | 27 | 7906 | 5.625830e-02 | 1.881465 | 0.011792 | ACOT7//ACOX1//ELOVL4//ELOVL7//SCD5 |
| hsa05414 | Dilated cardiomyopathy (DCM) - Homo sapiens (human) | http://www.genome.jp/kegg-bin/show\_pathway?hsa05414+70+109+153+3479+3678+3655+3685+3696+5567+7042+7124 | 1.336603e-02 | 11 | 424 | 96 | 7906 | 5.650955e-02 | 1.873998 | 0.025943 | ACTC1//ADCY3//ADRB1//IGF1//ITGA5//ITGA6//ITGAV//ITGB8//PRKACB//TGFB2//TNF |
| hsa04713 | Circadian entrainment - Homo sapiens (human) | http://www.genome.jp/kegg-bin/show\_pathway?hsa04713+109+805+2776+10681+2890+3760+5594+8864+5567+51655+9252 | 1.437547e-02 | 11 | 424 | 97 | 7906 | 6.001759e-02 | 1.842378 | 0.025943 | ADCY3//CALM2//GNAQ//GNB5//GRIA1//KCNJ3//MAPK1//PER2//PRKACB//RASD1//RPS6KA5 |
| hsa05100 | Bacterial invasion of epithelial cells - Homo sapiens (human) | http://www.genome.jp/kegg-bin/show\_pathway?hsa05100+23607+1398+1399+2549+3678+4233+5291+8503+8976 | 1.551940e-02 | 9 | 424 | 73 | 7906 | 6.399357e-02 | 1.809125 | 0.021226 | CD2AP//CRK//CRKL//GAB1//ITGA5//MET//PIK3CB//PIK3R3//WASL |
| hsa04914 | Progesterone-mediated oocyte maturation - Homo sapiens (human) | http://www.genome.jp/kegg-bin/show\_pathway?hsa04914+109+8697+64506+132864+22849+80315+3479+5594+5291+8503+5567 | 1.656570e-02 | 11 | 424 | 99 | 7906 | 6.747491e-02 | 1.780790 | 0.025943 | ADCY3//CDC23//CPEB1//CPEB2//CPEB3//CPEB4//IGF1//MAPK1//PIK3CB//PIK3R3//PRKACB |
| hsa05219 | Bladder cancer - Homo sapiens (human) | http://www.genome.jp/kegg-bin/show\_pathway?hsa05219+1026+1869+1871+5594+4313+9252 | 2.095749e-02 | 6 | 424 | 41 | 7906 | 8.433496e-02 | 1.678661 | 0.014151 | CDKN1A//E2F1//E2F3//MAPK1//MMP2//RPS6KA5 |
| hsa05410 | Hypertrophic cardiomyopathy (HCM) - Homo sapiens (human) | http://www.genome.jp/kegg-bin/show\_pathway?hsa05410+70+1906+3479+3678+3655+3685+3696+5562+7042+7124 | 2.178104e-02 | 10 | 424 | 90 | 7906 | 8.660555e-02 | 1.661921 | 0.023585 | ACTC1//EDN1//IGF1//ITGA5//ITGA6//ITGAV//ITGB8//PRKAA1//TGFB2//TNF |
| hsa05169 | Epstein-Barr virus infection - Homo sapiens (human) | http://www.genome.jp/kegg-bin/show\_pathway?hsa05169+10018+894+9134+1021+1026+1869+1871+1647+3439+10379+3716+3725+6416+5291+8503+864+6774+7124 | 2.226518e-02 | 18 | 424 | 201 | 7906 | 8.748908e-02 | 1.652374 | 0.042453 | BCL2L11//CCND2//CCNE2//CDK6//CDKN1A//E2F1//E2F3//GADD45A//IFNA1//IRF9//JAK1//JUN//MAP2K4//PIK3CB//PIK3R3//RUNX3//STAT3//TNF |
| hsa04625 | C-type lectin receptor signaling pathway - Homo sapiens (human) | http://www.genome.jp/kegg-bin/show\_pathway?hsa04625+805+1959+3558+3659+10379+3725+5594+5291+8503+5534+7124 | 2.312550e-02 | 11 | 424 | 104 | 7906 | 8.981300e-02 | 1.635909 | 0.025943 | CALM2//EGR2//IL2//IRF1//IRF9//JUN//MAPK1//PIK3CB//PIK3R3//PPP3R1//TNF |
| hsa04071 | Sphingolipid signaling pathway - Homo sapiens (human) | http://www.genome.jp/kegg-bin/show\_pathway?hsa04071+2776+4217+5594+8439+5291+8503+5515+5728+1901+81537+56848+7124 | 2.541550e-02 | 12 | 424 | 119 | 7906 | 9.757216e-02 | 1.594901 | 0.028302 | GNAQ//MAP3K5//MAPK1//NSMAF//PIK3CB//PIK3R3//PPP2CA//PTEN//S1PR1//SGPP1//SPHK2//TNF |
| hsa04152 | AMPK signaling pathway - Homo sapiens (human) | http://www.genome.jp/kegg-bin/show\_pathway?hsa04152+79602+3479+5214+5291+8503+5468+5515+5562+6009+79966+6517+7248 | 2.692706e-02 | 12 | 424 | 120 | 7906 | 1.022004e-01 | 1.569811 | 0.028302 | ADIPOR2//IGF1//PFKP//PIK3CB//PIK3R3//PPARG//PPP2CA//PRKAA1//RHEB//SCD5//SLC2A4//TSC1 |
| hsa04664 | Fc epsilon RI signaling pathway - Homo sapiens (human) | http://www.genome.jp/kegg-bin/show\_pathway?hsa04664+2885+6416+5594+5291+8503+6654+6655+7124 | 2.820959e-02 | 8 | 424 | 68 | 7906 | 1.058652e-01 | 1.549603 | 0.018868 | GRB2//MAP2K4//MAPK1//PIK3CB//PIK3R3//SOS1//SOS2//TNF |
| hsa04962 | Vasopressin-regulated water reabsorption - Homo sapiens (human) | http://www.genome.jp/kegg-bin/show\_pathway?hsa04962+109+10671+1783+140735+4905+5567 | 2.878087e-02 | 6 | 424 | 44 | 7906 | 1.068090e-01 | 1.540896 | 0.014151 | ADCY3//DCTN6//DYNC1LI2//DYNLL2//NSF//PRKACB |
| hsa04066 | HIF-1 signaling pathway - Homo sapiens (human) | http://www.genome.jp/kegg-bin/show\_pathway?hsa04066+1026+1906+54583+112399+3479+5594+2872+5214+5291+8503+6774 | 3.140870e-02 | 11 | 424 | 109 | 7906 | 1.152803e-01 | 1.502950 | 0.025943 | CDKN1A//EDN1//EGLN1//EGLN3//IGF1//MAPK1//MKNK2//PFKP//PIK3CB//PIK3R3//STAT3 |
| hsa05162 | Measles - Homo sapiens (human) | http://www.genome.jp/kegg-bin/show\_pathway?hsa05162+894+9134+1021+3439+3558+10379+3716+3725+4478+5291+8503+51209+6774 | 3.357250e-02 | 13 | 424 | 138 | 7906 | 1.218828e-01 | 1.474016 | 0.030660 | CCND2//CCNE2//CDK6//IFNA1//IL2//IRF9//JAK1//JUN//MSN//PIK3CB//PIK3R3//RAB9B//STAT3 |
| hsa04390 | Hippo signaling pathway - Homo sapiens (human) | http://www.genome.jp/kegg-bin/show\_pathway?hsa04390+659+894+122786+11211+8323+26524+4771+5515+83439+7003+7042+7048+7476+7532 | 3.654075e-02 | 14 | 424 | 154 | 7906 | 1.312324e-01 | 1.437223 | 0.033019 | BMPR2//CCND2//FRMD6//FZD10//FZD6//LATS2//NF2//PPP2CA//TCF7L1//TEAD1//TGFB2//TGFBR2//WNT7A//YWHAG |
| hsa01524 | Platinum drug resistance - Homo sapiens (human) | http://www.genome.jp/kegg-bin/show\_pathway?hsa01524+1026+2939+4217+5594+5291+8503+5366+5980 | 4.078739e-02 | 8 | 424 | 73 | 7906 | 1.441946e-01 | 1.389474 | 0.018868 | CDKN1A//GSTA2//MAP3K5//MAPK1//PIK3CB//PIK3R3//PMAIP1//REV3L |
| hsa05167 | Kaposi sarcoma-associated herpesvirus infection - Homo sapiens (human) | http://www.genome.jp/kegg-bin/show\_pathway?hsa05167+805+1021+1026+1869+1871+10681+3439+10379+3716+3725+6416+5594+5291+8503+5534+6774 | 4.127892e-02 | 16 | 424 | 186 | 7906 | 1.441946e-01 | 1.384272 | 0.037736 | CALM2//CDK6//CDKN1A//E2F1//E2F3//GNB5//IFNA1//IRF9//JAK1//JUN//MAP2K4//MAPK1//PIK3CB//PIK3R3//PPP3R1//STAT3 |
| hsa04024 | cAMP signaling pathway - Homo sapiens (human) | http://www.genome.jp/kegg-bin/show\_pathway?hsa04024+51+109+153+805+1129+1812+1906+2890+610+3725+5594+10846+5291+8503+4659+5567+5908+11069 | 4.144517e-02 | 18 | 424 | 216 | 7906 | 1.441946e-01 | 1.382526 | 0.042453 | ACOX1//ADCY3//ADRB1//CALM2//CHRM2//DRD1//EDN1//GRIA1//HCN2//JUN//MAPK1//PDE10A//PIK3CB//PIK3R3//PPP1R12A//PRKACB//RAP1B//RAPGEF4 |
| hsa04916 | Melanogenesis - Homo sapiens (human) | http://www.genome.jp/kegg-bin/show\_pathway?hsa04916+109+805+1906+11211+8323+2776+5594+5567+83439+7476 | 4.369716e-02 | 10 | 424 | 101 | 7906 | 1.504624e-01 | 1.359547 | 0.023585 | ADCY3//CALM2//EDN1//FZD10//FZD6//GNAQ//MAPK1//PRKACB//TCF7L1//WNT7A |
| hsa04960 | Aldosterone-regulated sodium reabsorption - Homo sapiens (human) | http://www.genome.jp/kegg-bin/show\_pathway?hsa04960+3479+5594+4306+5291+8503 | 4.591703e-02 | 5 | 424 | 37 | 7906 | 1.564927e-01 | 1.338026 | 0.011792 | IGF1//MAPK1//NR3C2//PIK3CB//PIK3R3 |
| hsa04062 | Chemokine signaling pathway - Homo sapiens (human) | http://www.genome.jp/kegg-bin/show\_pathway?hsa04062+109+6347+6355+1398+1399+9547+10681+2885+5594+5291+8503+5567+5908+6654+6655+6774 | 4.664403e-02 | 16 | 424 | 189 | 7906 | 1.573647e-01 | 1.331204 | 0.037736 | ADCY3//CCL2//CCL8//CRK//CRKL//CXCL14//GNB5//GRB2//MAPK1//PIK3CB//PIK3R3//PRKACB//RAP1B//SOS1//SOS2//STAT3 |
| hsa05235 | PD-L1 expression and PD-1 checkpoint pathway in cancer - Homo sapiens (human) | http://www.genome.jp/kegg-bin/show\_pathway?hsa05235+29126+3716+3725+5594+5291+8503+5534+5728+6774 | 4.831410e-02 | 9 | 424 | 89 | 7906 | 1.613691e-01 | 1.315926 | 0.021226 | CD274//JAK1//JUN//MAPK1//PIK3CB//PIK3R3//PPP3R1//PTEN//STAT3 |
